# CLE42 binding induces PXL2 interaction with SERK2

**DOI:** 10.1007/s13238-017-0435-1

**Published:** 2017-07-04

**Authors:** Shulin Mou, Xiaoxiao Zhang, Zhifu Han, Jiawei Wang, Xinqi Gong, Jijie Chai

**Affiliations:** 1 Innovation Center for Structural Biology, Tsinghua-Peking Joint Center for Life Sciences, School of Life Sciences, Tsinghua University, Beijing, 100084 China; 2 Institute for Mathematical Sciences, Renmin University of China, Beijing, 100872 China

**Dear Editor,**

Plant tissues derived from meristems including SAM (shoot apical meristem) and RAM (root apical meristem) are located at the tips of shoot and root and procambial cell tissues in the vascular system (Simon and Stahl, 2011). Through asymmetric periclinal cell division, a few layers of stem cells in vascular meristems differentiate into apposing xylem and phloem cells, forming the conducting system and playing an important role in long-distance transport of water, nutrients, sugars, and signaling molecules such as hormones in plant (Elo et al., 2013).

The leucine rich repeat receptor kinase (LRR-RK) PXY (phloem intercalated with xylem) belongs to XI subfamily of leucine rich repeat receptor-like kinase (LRR-RLK). PXY is a receptor of CLAVATA3/EMBRYO SURROUNDING REGION-RELATED (CLE) peptide CLE41/44 or TDIF (tracheary elements differentiation inhibitory factor). TDIF-PXY signaling functions to promote procambial cell proliferation and suppress tracheary element differentiation, thus playing an important role in wood formation and vascular (Hirakawa et al., 2008; Ito et al., 2006; Fisher and Turner, 2007). The *tdr-1/pxy-5* mutant was severely impaired in the proliferation of procambial cells (Hirakawa et al., 2008). WUSCHEL HOMEOBOX RELATED 4 (WOX4) and WOX14 are downstream components of PXY-TDIF signaling and function redundantly in regulating vascular cell division (Etchells et al., 2013; Hirakawa et al., 2010). CLE41/44 has 12 aa (His-Glu-Val-Hyp-Ser-Gly-Hyp-Asn-Pro-Ile-Ser-Asn) in its mature form. A recent structural study revealed that the last amino acid of the peptide is required for CLE41/44 recognition by PXY (Zhang et al., 2016b).

PXL1 (PXY-like 1) and PXL2 (PXY-like 2) are two closely related LRR-RKs to PXY, sharing 61% and 62% sequence similarity with PXY, respectively. However, in contrast with PXY, neither *pxl1* nor *pxl2* plants displayed an obvious phenotype in the vascular stem (Fisher and Turner, 2007). Nonetheless, simultaneous mutations of the three LRR-RKs genes (*pxy-3* with *pxl1* and *pxl2*) generated an enhanced vascular phenotype observed in *pxy-3* plants with flatter vascular bundles and a less clear distinction between xylem and phloem. These results suggest that *PXL1* and *PXL2* can function redundantly or synergistically with *PXY* in regulating vascular-tissue development. Indeed, biochemical data showed that CLE41/44 also interacted with PXL1, though with a lower affinity than that of CLE41/44 with PXY (Zhang et al., 2016b). Interestingly, the triple-mutant did not display a more pronounced phenotype than the *pxl1* and *pxl2* plants, suggesting that these two genes might also have a different role from PXY in vascular development (Fisher and Turner, 2007).

PXL2 belongs to XI LRR-RK subfamily, members of which have been proposed to recognize small signaling peptides through the conserved Arg-x-Arg (RxR, x stands for any amino acid) motif (Zhang et al., 2016a). We therefore reasoned that PXL2 may also recognize a small signaling peptide(s) to mediate vascular development. To test this idea, we purified the extracellular LRR domain protein of PXL2 (PXL2^LRR^) and incubated the purified protein with a pool of chemically synthesized peptides featuring a free C-terminal histidine or asparagine. The mixture was then subject to gel filtration to separate the PXL2^LRR^-bound peptide(s) from the others (Fig. 1A). The protocol described previously (Song et al., 2016) was used to detect the peptide(s) bound to the PXL2^LRR^ protein by mass spectrometry. By using this method, we found that CLE42 was co-purified with the PXL2^LRR^ protein in the gel filtration assay, suggesting that CLE42 may act as a ligand of PXL2 (Fig. 1B). To further support this conclusion, we assayed the binding affinity of CLE42 with PXL2^LRR^ using ITC. The ITC results showed that CLE42 bound to the PXL2^LRR^ protein with a dissociation constant (*K*_d_) of ~2.75 μmol/L (Fig. 1C). CLE42 is also a dodecapeptide (His-Gly-Val-Hyp-Ser-Gly-Hyp-Asn-Pro-Ile-Ser-Asn) and differs from CLE41 only in the 2nd position. ITC assays indicated that CLE41 also interacted with PXL2^LRR^ but with a slightly lower affinity (*K*_d_ ~10 μmol/L, Fig. 1D). As a negative control, CLE13 (Arg-Leu-Val-Hyp-Ser-Gly-Hyp-Asn-Pro-Leu-His-His) had no detectable interaction with PXL2^LRR^ as indicated by ITC (Fig. S1).

**Figure 1:**
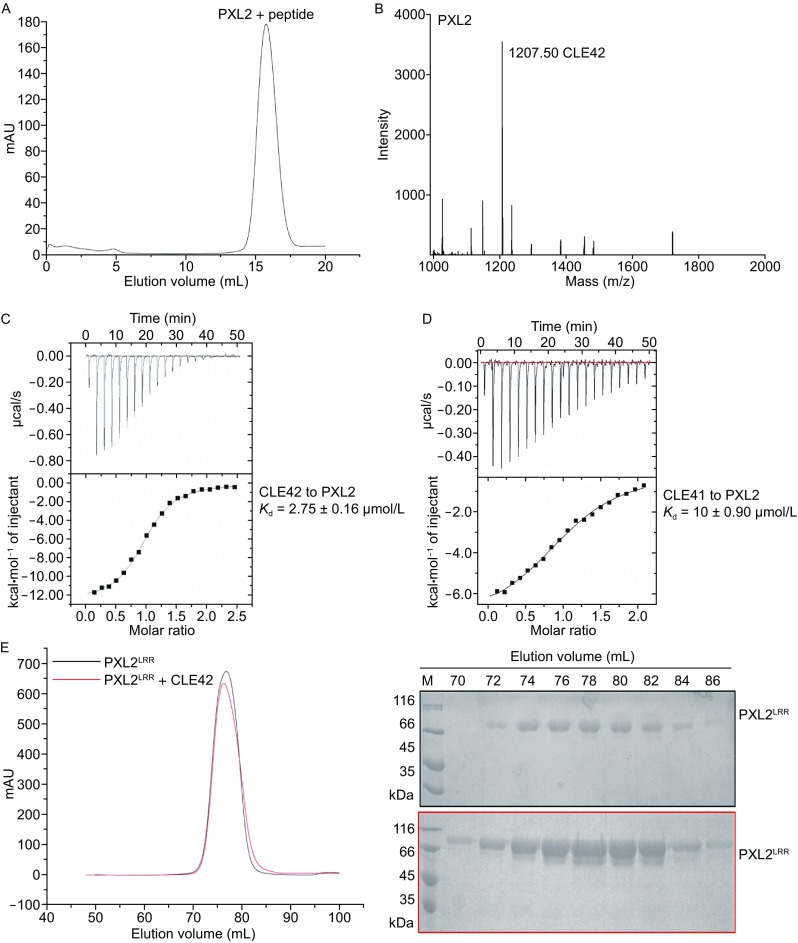

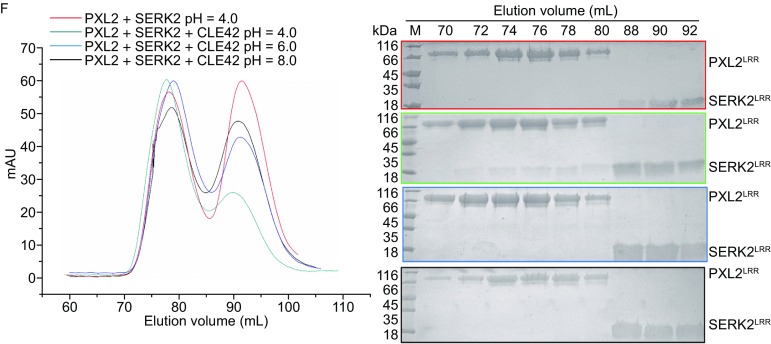
**CLE42 binding induces PXL2** ^**LRR**^ **interaction with SERK2** ^**LRR**^. (A) Gel-filtration chromatogram of the extracellular LRR domain protein of PXL2 (PXL2^LRR^) and a pool of synthesized peptides. The peak indicates the elution positions of PXL2^LRR^-peptide in gel filtration. The vertical and horizontal axes represent UV absorbance (280 nm) and elution volume (mL) respectively. (B) MALDI-TOF MS of the peak fraction of PXL2^LRR^-peptide shown in (A). The molecular weight of the peptide from the peak fraction (1207.50) indicated is equivalent to the theoretical weight of CLE42. The vertical and horizontal axes represent the intensity and molecular weight of MS respectively. (C) Measurement of the binding affinity between PXL2^LRR^ and CLE42 by ITC. Top panel: twenty injections of CLE42 solution were titrated into PXL2^LRR^ in the ITC cell. The area of each injection peak corresponds to the total heat released for that injection. Bottom panel: the binding isotherm for PXL2^LRR^-CLE42 interaction. The integrated heat is plotted against the molar ratio between CLE42 and PXL2^LRR^. Data fitting revealed a binding affinity of about 2.75 μmol/L. (D) Measurement of binding affinity between PXL2^LRR^ and CLE41/TDIF by ITC. The assay was performed as described in (C). Data fitting revealed a binding affinity of about 10 μmol/L. (E) CLE42 binding induces no oligomerization of PXL2^LRR^. Left: gel filtration profiles of PXL2^LRR^ in the presence and absence of CLE42. The vertical and horizontal axes represent ultraviolet absorbance (λ = 280 nm) and elution volume (mL), respectively. Right: Coomassie blue staining of the peak fractions of PXL2^LRR^ shown in the left following SDS-PAGE. M, molecular weight ladder (kDa). (F) CLE42 induces PXL2^LRR^-SERK2^LRR^ heterodimerization in solution at pH 4.0. Top panel, gel filtration profiles of PXL2^LRR^ and SERK2^LRR^ in the presence (slate at pH 4.0, blue at pH 6.0, black at pH 8.0), and absence (red) of CLE42. Right: Coomassie blue staining of the peak fractions of PXL2^LRR^ and SERK2^LRR^ shown in (F) following SDS–PAGE

We then solved the crystal structure of PXL2^LRR^ determined at resolution of 3.6 Å (Fig. 2A). Structural comparison showed that PXL2^LRR^ and PXY^LRR^ are highly conserved in their structures (Fig. S2A). Although we have not obtained the structure of PXL2^LRR^ bound by CLE42, the complex can be modeled with high confidence using the structure of PXY^LRR^-CLE41 as a template given that the conserved structures of PXL2^LRR^ and PXY^LRR^ and high sequence identity between CLE41 and CLE42 (Fig. 2B). In the modeled structure of PXL2^LRR^-CLE42, the small peptide also adopts an “Ω”-like kink to interact with PXL2, forming a set of interactions (Fig. 2C–E) conserved in the PXY^LRR^-CLE41 interaction. The total buried surface areas generated by CLE42 binding to PXL2^LRR^ and CLE41 to PXY^LRR^ are similar to each other. However, the non-polar buried surface area in the CLE42-PXL2^LRR^ complex (~762 Å^2^) is slightly larger than that in the CLE41-PXL2^LRR^ complex (~726 Å^2^), affording an explanation for our observations that the former is a tighter complex than the latter. Interestingly, the ratio between the non-polar buried surfaces of PXL2^LRR^-CLE41 and PXL2^LRR^-CLE42 (0.95) is close to that of the logarithms of the experimental *K*_d_ values of PXL2^LRR^-CLE42 (2.75 μmol/L) and PXL2^LRR^-CLE41 (10.00 μmol/L). These observations are consistent with the proposed relationship between non-polar buried interfacial area and binding affinity (Chen et al., 2013) (Table 1).

**Figure 2:**
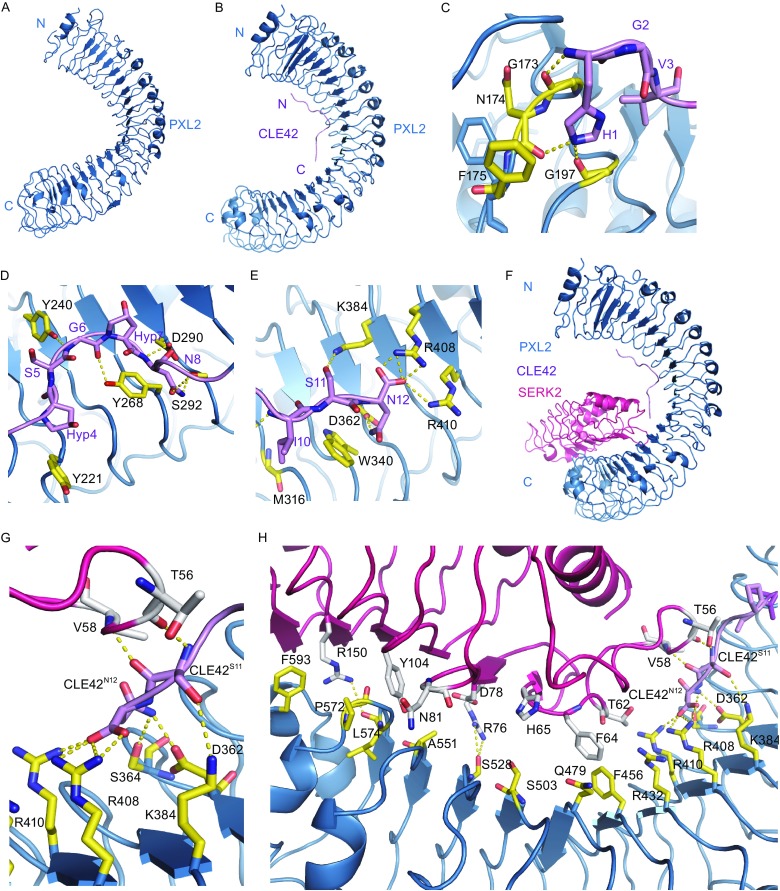
**Modeled structure of CLE42-PXL2** ^**LRR**^ and PXL2^LRR^-CLE42-SERK2^**LRR**^. (A) Crystal structure of PXL2^LRR^ alone shown in cartoon. (B) Modeled structure of PXL2^LRR^-CLE42 complex using as the PXY^LRR^-CLE41 template. CLE42 adopts an “Ω”-like kink and binds to the concave surface of PXL2^LRR^. (C) Detailed interaction of the N-terminal side of CLE42 with PXL2^LRR^. The side chains of some amino acids from CLE42 and PXL2^LRR^ are shown in violet and yellow orange, respectively. Yellow dashed lines indicate hydrogen bonds or salt bridges. (D) Detailed interaction of the central region of CLE42 with PXL2^LRR^. (E) Detailed interaction of the C-terminal side of CLE42 with PXL2^LRR^. (F) Model structure of PXL2^LRR^-CLE42-SERK2^LRR^ complex. (G) Detailed interaction between the C-terminal residues Ser11 and Asn12 of CLE42 and PXL2, SERK2. The side chains of some amino acids from CLE42, PXL2^LRR^, and SERK2^LRR^ are shown in violet, yellow orange, and pink, respectively. Yellow dashed lines indicate hydrogen bonds or salt bridges. (H) Detailed interaction between PXL2^LRR^-CLE42 and SERK2^LRR^

**Table 1:** Datacollection and refinement statistics

	PXL2
Wavelengh (Å)	0.9790
Resolution range (Å)	108.37–3.60 (3.71–3.60)
Space group	P2_1_2_1_2
Unit-cell	128.05, 203.42, 68.49
	90.00,90.00,90.00
Unique reflections	18571 (2891)
Completeness (%)	99.98 (87.2)
Mean I/sigma (I)	13.6 (3.2)
Redundancy	3.0 (3.1)
*R*_sym_ (%)	15.7 (64.2)
*R*_work_	0.277 (0.423)
*R*_free_	0.321 (0.523)
R.m.s.d (bonds)	0.006
R.m.s.d (angels)	2.038

* ${\text{Rsym}} = \sum\nolimits_{hkl} {\sum\nolimits_{i} {\left| {\text{(}hkl\text{)}} \right.} } { - }\left\langle {I(hkl)} \right\rangle \left| {/\sum\nolimits_{hkl} {\sum\nolimits_{i} {Ii} } } \right.(hkl)$ where Ii(hkl) is the intensity of the ith observation of reflection hkl and 〈I(hkl)〉 is the average over all observations of reflection hkl

In the structure of the CLE41-SERK2^LRR^-PXY^LRR^ complex (Zhang et al., 2016c), the C-terminal side of CLE41 forms a pair of hydrogen bonds with SERK2, thus contributing to the interaction between SERK2^LRR^ and PXY^LRR^. Structural comparison between this complex and CLE42-SERK2^LRR^-PXL2^LRR^ (the modeled structure) showed that the C-terminal portions of the two small peptides are highly conserved in their receptor-bound forms (Fig. 2F–H). This result suggests that PXL2 may also use SERK member as a co-receptor if CLE42 indeed function as a ligand of PXL2. To test this idea, we first assayed that the PXL2^LRR^ protein in the presence or absence of CLE42. As shown in Figure 1E, CLE42 binding induce no oligoimerization of PXL2^LRR^, because the elution volume of the protein did not change in the presence of CLE42, suggesting that a co-receptor is required for CLE42-induced signaling based on the dimerization model (Han et al., 2014). To test whether SERK members are able to form CLE42-induced complexes with PXL2, we purified the extracellular LRR domain protein of SERK2 (SERK2^LRR^) and used gel filtration to examine its interaction with the purified PXL2^LRR^ protein in the presence of the chemically synthesized CLE42. Indeed, the gel-filtration results showed that SERK2 protein formed a stable complex with PXL2^LRR^ in the presence but not in the absence of CLE42 when the assays were performed at an acidic pH (pH = 4.0) (Fig. 1F). Like other small peptide-induced interaction between a SERK member and an LRR-RK (Sun et al., 2013), the CLE42 induced SERK2^LRR^-PXL2^LRR^ interaction was pH-dependent, as increasing pH to 6.0 or 8.0 resulted in non-detectable interaction between SERK2^LRR^-PXL2^LRR^ even in the presence of CLE42 (Fig. 1F).

Here we provide biochemical evidence showing that CLE42 interacts with PXL2 *in vitro*. Consistent with CLE42 as a ligand of PXL2, we also showed that CLE42 induced interaction with of PXL2 and the SERK family member SERK2. However, the biological functions of these interactions still remain unknown. A role of CLE42 in suppressing xylem formation has been shown before (Hirakawa et al., 2008). CLE42 is expressed strongly in shoot apical meristem (SAM) and axillary meristems to enhance axillary bud formation (Yaginuma et al., 2011). Consistently, excess formation and outgrowth of axillary buds has been shown in plants overexpressing CLE41 and CLE42. However, mutation of TDR did not completely suppress the promotion of axillary bud formation by CLE42 peptide, suggesting that other receptor(s) might exist for perception of the two peptides. Based on the biochemical data reported here, we propose that PXL2 may function as a receptor of CLE42 and probably CLE41 as well. However, PXL2, also called MIK1 (MDIS1-INTERACTING RECEPTOR LIKE KINASE1), was recently shown to form heteromers with MDISI (MALE DISCOVERER1) and perceive the female attractant peptide LURE1 in *Arabidopsis* thaliana (Wang et al., 2016). One explanation to reconcile our biochemical data with these genetic data is that PXL2/MIK1 can serve as a dual receptor of different ligands, thus mediate different peptide-induced signaling. The same LRR-RK that can perceive two different ligands has been reported (Deyoung and Clark, 2008; Shinohara et al., 2012). Furthermore, a role of PXL2 in vascular tissue development is also in line with the genetic data showing that simultaneous mutations of the three LRR-RKs genes (*pxy-3* with *pxl1* and *pxl2*) generate a more striking vascular phenotype as compared to the *pxy-3* plants. Nonetheless, future studies are needed to investigate whether PXL2 function as a receptor of CLE42 to mediate plant vascular tissue development.

## Electronic supplementary material

Below is the link to the electronic supplementary material.

Supplementary material 1 (PDF 154 kb)

Supplementary material 2 (EPS 660 kb)

Supplementary material 3 (EPS 2269 kb)

Supplementary material 4 (EPS 4440 kb)
