# Supplementary material for: CLE42 binding induces PXL2 interaction with SERK2
Source: Protein Cell. 2017 Jul 4;8(8):612–7. doi: 10.1007/s13238-017-0435-1 (PMC5546936; doi:10.1007/s13238-017-0435-1)
Supplement: Supplementary file 1 — Supplementary material 1 (PDF 154 kb) [file 13238_2017_435_MOESM1_ESM.pdf]

MATERIALS AND METHODS

Protein expression and purification

The LRR domain of PXL2 (residues 1-633, $PXL2^{LRR}$) from Arabidopsis with an engineered C-terminal 6×His-tag was generated by standard PCR based cloning strategy and its identity was confirmed by sequencing. The protein was expressed in High Five cells using the vector pFastBac 1 (Invitrogen). One litre of cells ($1.8×10^6$ cells/ml cultured in the medium from Expression Systems) was infected with 20 mL baculovirus at 22℃, and protein was harvested from the media after 48 h. The protein was purified using Ni-NTA (Novagen) and size-exclusion chromatography (Hiload 200, GE Healthcare) in buffer containing 10 mM BisTris pH 6.0, 100 mM sodium chloride at 4℃. For crystallization of $PXL2^{LRR}$, the purified protein was concentrated to about 10 mg/ml.

Gel fltration and MS assay

Approximately 0.5 mg of purifed $PXL2^{LRR}$ domain proteins (in 1.0 ml buffer containing 10 mM Bis-Tris pH 6.0, 100 mM NaCl) were incubated with a peptide pool (0.1mg for each) on ice for 1 h. The peptide pool are some CLE and CLE-Like peptides .Then the mixture was analyzed by gel fltration (Superdex200, GE Healthcare), the peak fraction was collected and 10 µl was used for MALDI TOF-MS.

Crystallization, data collection, structure determination and refinement

The crystals of $PXL2^{LRR}$ were obtained at 18 ℃ by the hanging-drop vapor-diffusion method. The $PXL2^{LRR}$ protein was crystallized by mixing 1 µl of the protein and reservoir solution containing 0.1 M Sodium citrate tribasic dihydrate pH 5.5 and 22% w/v Polyethylene glycol 1,000. To prevent the crystals from radiation damage, all crystals were flash frozen using the reservoir buffer plus 20% glycerol as the cryoprotectant. Crystals grew to their maximum size ($0.1 × 0.1 × 0.05\ mm^3$) within 30 days at 18 ℃. The diffraction data were collected at Shanghai Synchrotron Radiation Facility (SSRF) on the beam line BL17U1. All the data were processed using HKL2000 software package. Molecular replacement (MR) with the program PHASER (McCoy et al., 2007) was used to solve the crystal structure of $PXL2^{LRR}$. The atomic coordinates of PXY (PDB ID: 5GIJ) were used as the initial searching model. The model from MR was built with the program COOT (Emsley and Cowtan, 2004) and subsequently subjected to refinement by the program PHENIX (Adams et al., 2002). The statistics of the data collection and refinement for the structure is shown in Table S1. The structure figures were prepared using PyMOL (http://www.pymol.org)

Structural modeling of the $PXL2^{LRR}$-CLE41/CLE42 and $PXL2^{LRR}$ -CLE42 -$SERK2^{LRR}$

The complex formed by the interaction between PXL2- CLE42/CLE41 and $PXL2^{LRR}$ -CLE42

-SERK2^{LRR} were predicted by the docking method HoDock (Gong et al., 2010) which incorporates an initial rigid docking and a refined semi-flexible docking. In this work, the experimental solved structure PXY-CLE41 and PXY-CLE41-SERK2 showing a promisingly similar binding mode, which were both used as restraints for conformational searching and model selection. Totally 35,000 complex structures were generated and scored to pick up the final correct complex structure model.

ITC assay

The binding affinities of PXL2^{LRR} with CLE13/41/42 were measured using MicroCalorimeter ITC200 (Microcal LLC) at 25 °C in the buffer containing 10 mM Bis-Tris, pH 6.0, and 100 mM NaCl. Approximately 0.5 mM CLE13/41/42 was injected into the stirred calorimeter cell (300 µl) containing PXL2^{LRR} (0.05mM) with 20 × 2 µl at 2.5-min intervals. The stirring speed was 750 r.p.m.. The heat of dilution from titration of a peptide into the buffer was subtracted. Each experiment was repeated at least three times. All the titration data were analyzed using the ORIGIN software (MicroCal Software).

Adams PD, Grosse-Kunstleve RW, Hung LW, et al. PHENIX:building new software for automated crystallographic structure determination. Acta Crystallogr D Biol Crystallogr 2002; 58:1948-1954.

Emsley P, Cowtan K. Coot: model-building tools for molecular graphics. Acta Crystallogr D Biol Crystallogr 2004; 60:2126-2132.

Gong, X., Wang, P., Yang, F., Chang, S., Liu, B., He, H., Cao, L., Xu, X., Li, C., and Chen, W. (2010). Protein-protein docking with binding site patch prediction and network-based terms enhanced combinatorial scoring. Proteins 78, 3150-3155.

McCoy AJ, Grosse-Kunstleve RW, Adams PD, Winn MD,Storoni LC, Read RJ. Phaser crystallographic software. J Appl Crystallogr 2007; 40:658-674.

Fig. S1 **CLE13 displays no interaction with PXL2^{LRR}**

Measurement of binding affinity between PXL2^{LRR} and CLE13 by ITC. Twenty injections of CLE13 solution were titrated into PXL2^{LRR} in the ITC cell. The area of each injection peak corresponds to the total heat released for that injection. Bottom panel: the binding isotherm for PXL2^{LRR}-CLE13 interaction. The data showed no interaction between CLE13 and PXL2^{LRR}.

Fig. S2 **Structure comparison between PXY^{LRR} and PXL2^{LRR}**

(A) Structural superposition of the PXY^{LRR} (wheat) and PXL2^{LRR} (sky blue).

(B) The two consecutive LRRs recognizing the last residue of CLE42 are stabilized by a conserved disulfide bond. The sulfur atoms are colored in cyan. The side chains of some amino acids from CLE42 and PXL2^{LRR} are shown in violet and yellow orange, respectively. Yellow dashed lines indicate hydrogen bonds or salt bridges.

Fig. S3 **Modeled structure of PXL2^{LRR}-CLE41 complex**

(A) Modeled structure of PXL2^{LRR}-CLE41 complex shown cartoon.

(B) Interaction of CLE41 with PXL2$^{LRR.}$ The side chains of some amino acids from

CLE41 and $PXL2^{LRR}$ are shown in palm green and yellow orange, respectively.
